# Structure-Related Mechanical Properties and Bioactivity of Silica–Gelatin Hybrid Aerogels for Bone Regeneration

**DOI:** 10.3390/gels9010067

**Published:** 2023-01-14

**Authors:** María V. Reyes-Peces, Rafael Fernández-Montesinos, María del Mar Mesa-Díaz, José Ignacio Vilches-Pérez, Jose Luis Cárdenas-Leal, Nicolás de la Rosa-Fox, Mercedes Salido, Manuel Piñero

**Affiliations:** 1 Departamento de Física de la Materia Condensada, Facultad de Ciencias, Universidad de Cádiz, 11510 Puerto Real, Spain; 2 Departamento de Histología, SCIBM, Facultad de Medicina, Universidad de Cádiz, 11004 Cádiz, Spain; 3 Instituto de Biomedicina de Cádiz, (INIBICA), Universidad de Cadiz, 11510 Puerto Real, Spain; 4 Departamento de Ingeniería Química, Facultad de Ciencias, Universidad de Cádiz, 11510 Puerto Real, Spain; 5 Instituto de Microscopía Electrónica y Materiales (IMEYMAT), Universidad de Cadiz, 11510 Puerto Real, Spain; 6 Departamento de Física Aplicada, Escuela Superior de Ingeniería, Universidad de Cádiz, 11510 Puerto Real, Spain

**Keywords:** gelatin, GPTMS, hybrid aerogel, percolation threshold, bioactivity, cell adhesion, osteoblasts, bone tissue engineering, mineralization, cytoskeleton

## Abstract

We report the synthesis of mesoporous silica–gelatin hybrid aerogels with 15, 25, and 30 wt. % gelatin contents, using 3-glycidoxypropyl trimethoxysilane (GPTMS) as a coupling agent, for tissue-engineering applications. Aerogels were obtained using a one-step sol–gel process followed by CO_2_ supercritical drying, resulting in crack-free monolith samples with bulk densities ranging from 0.41 g cm^−3^ to 0.66 g cm^−3^. Nitrogen adsorption measurements revealed an interconnected mesopore network and a general decrease in the textural parameters: specific surface areas (651–361 m^2^ g^−1^), pore volume (1.98–0.89 cm^3^ g^−1^), and pore sizes (10.8–8.6 nm), by increasing gelatin content. Thermogravimetric analysis (TGA), Fourier-transform infrared (FTIR) spectroscopy and uniaxial compression experiments confirmed that the structure, thermal properties and mechanical behavior of these aerogels changed significantly when the concentration of gelatin reached 25 wt.%, suggesting that this composition corresponds to the percolation threshold of the organic phase. In addition, the samples exhibited hydrophilic behavior and extremely fast swelling in phosphate-buffered saline (PBS), with swelling ratios from 2.32 to 3.32. Furthermore, in vitro bioactivity studies revealed a strong relationship between the kinetics of the nucleation and growth processes of hydroxyapatite in simulated body fluid (SBF) and the gelatin content. The live/dead assay revealed no cytotoxicity in HOB^®^ osteoblasts in vitro and a positive influence on cell growth, focal adhesion development, and cytoskeletal arrangement for cell adhesion. Mineralization assays confirmed the positive effects of the samples on osteoblast differentiation. The biomaterials described are versatile, can be easily sterilized and are suitable for a wide range of applications in bone tissue-engineering, either alone or in combination with bioactive-reinforced phases.

## 1. Introduction

Silica biopolymer mesoporous aerogels have been widely used in a variety of environmental and biomedical applications, such as oil recovery or heavy metal ion biosorbents for wastewater treatment [1,2], controlled drug release [3,4,5], and as biomaterials in regenerative biomedicine [6,7,8]. Therefore, considerable effort has been devoted to the synthesis of nanostructured bio-based silica aerogels by mixing silicate precursors with different biopolymers, usually polysaccharides such as chitosan [9,10], alginate [11], pectin [12], cellulose [13], or proteins as collagen [14] and gelatin [15]. Among these bio-based nanostructures, silica–gelatin (denaturalized collagen II) aerogels have attracted much attention in biomedical engineering because they can exhibit suitable properties by choosing appropriate crosslinker agents, thus enabling precise control of degradation rates and mechanical properties [3,6,7,16]. In addition, this type of hybrids have demonstrated to induce in vitro biomineralization in simulated body fluid (SBF), as well as excellent cell adhesion [17].

Many methods have been used to prepare silica–gelatin aerogels, usually using sol–gel techniques followed by the removal of the solvent within the hybrid porous network through supercritical drying [5,18] ambient drying [19] or freeze-drying [6]. Thus far, various crosslinker agents, mainly organosilanes, have been used to functionalize gelatin intending to develop class II hybrid materials with covalent coupling between the organic and inorganic networks [20], including 3-glycidoxypropyltrimethoxysilane (GPTMS) [7,21,22], 3-aminopropyltriethoxysilane (APTES) [23] and 3-aminopropyltrimethoxysilane (APTMS) [8]. Thus, it has been shown that biocompatible silica–GPTMS–gelatin materials, prepared by combining the sol–gel foaming process with freeze-drying, enable the generation of highly porous scaffold structures with tailorable material properties by varying the gelatin content and the degree of GPTMS crosslinking [7]. More recently, drug delivery mesoporous aerogel matrices based on a silica–APTMS–gelatin system have been processed with supercritical drying in CO_2_, exhibiting biocompatibility as a potential platform for cancer therapy [4]. Likewise, many other silica–gelatin aerogel materials designed for specific applications have been reported in the literature, combining a variety of synthetic strategies with different chemical precursors and crosslinker agents, as well as the type of the drying technique chosen to eliminate the liquid solvent from the wet gel [1,2,15,19,24,25,26,27].

In this paper, we report the synthesis and structural properties of new hybrid silica–gelatin-based aerogels synthesized from tetraethoxysilane (TEOS), GPTMS and different gelatin contents, and processed under CO_2_ supercritical drying. The use of GPTMS is based on the fact that it bonds with both nucleophilic functional groups of gelatin and silanol groups of the silica network [7,21]. Furthermore, it provides independent control over the mechanical and degradation properties of the resulting biomaterials, thereby promoting cell adhesion and proliferation [17]. The mechanical behavior of the samples and their bioactive responses in in vitro experiments in SBF were studied. The swelling behavior and absorption kinetics of the samples in PBS were also tested. To the best of our knowledge, studies on the structure-related mechanical properties and bioactive response of silica–gelatin hybrid porous materials crosslinked with GPTMS processed in supercritical CO_2_ are scarce, and this work aims to contribute to this research subject by investigating the results based on these types of silica–GPTMS–gelatin aerogels.

## 2. Results and Discussion

The procedure designed in this study was intended to fabricate homogeneous silica–gelatin hybrid aerogel monoliths with tailorable structural and mechanical properties for tissue regeneration. This system has been extensively studied in recent years, because gelatin is a degraded form of collagen, which is the most important protein in the extracellular matrix of all tissues and promotes good cell adhesion when incorporated into a variety of biomaterials [6]. To prevent fast disintegration and instability of gelatin in body fluids, previous researchers have proposed its functionalization to produce hybrid materials with silica, and the coupling agent most frequently used for gelatin-containing systems is GPTMS [28,29]. In this study, we were motivated by several synthetic routes previously proposed in the literature [6,30] where the silica contribution came from both GPTMS and a separate source of silica using TEOS. To obtain the highest degree of crosslinking in the resulting aerogel hybrids, functionalization of gelatin with GPTMS was performed without pH correction, to avoid the condensation reaction between the siloxane groups of GPTMS and the loss of its coupling effect when mixed with hydrolyzed TEOS. Additionally, the drying process to obtain the aerogel material was performed using supercritical CO_2_ instead of other methods such as freeze-drying. A more detailed description of the synthesis procedure for the obtained aerogels is provided in Section 4.1. In particular, gelatin/silica weight ratios of approximately 15, 25, and 30 wt. % were prepared, named SG15, SG25 and SG30 (see Table 1). For this calculation, the amount of silica formed was considered to be due to the hydrolysis and condensation of both TEOS and GPTMS precursors. Previous studies have suggested that this range of compositions provides appropriate gelatin network stability for biodegradation as well as improved bioactivity of the materials [6,18].

### 2.1. Physical and Textural Properties of the Hybrid Aerogels

**Figure 1:**
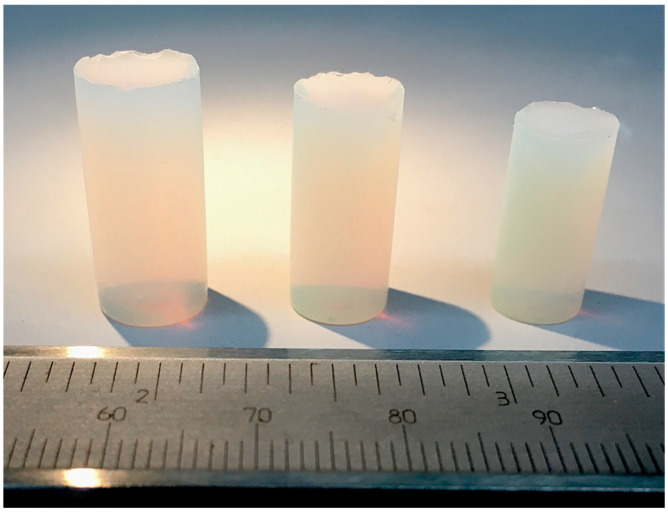
Representative sample set of SiO_2_–GPTMS–gelatin hybrid aerogels extracted from a supercritical drying CO_2_ autoclave. The aerogels were aligned by increasing gelatin content from left to right: 15 wt. %, 25 wt. %, and 30 wt. %.

Homogenous and elastic hybrid aerogels with different gelatin contents were obtained under supercritical drying in CO_2_ as monolith cylinders, measuring 15–22 mm in height and 8–10 mm in diameter (see Figure 1).

The influence of the protein content on the bulk density and volume shrinkage was studied, and the results are presented in Table 2. The well-defined geometry of the as-produced aerogels facilitated the estimation of both physical parameters, which were significantly affected by the progressive increase of gelatin, from ρ_a_ = 0.41 gcm^−3^ in the SG15 aerogel to ρ_a_ = 0.69 gcm^−3^ in the SG30 sample. This macroscopic trend observed in the density indicates an increase in the incorporation of the organic phase into the porous matrix. However, the bulk density measurements were considerably higher than those previously reported for similar types of aerogels composed of silica and gelatin [2]. Accordingly, an increase in gelatin content resulted in an increase in volume shrinkage of the hybrid aerogels after supercritical drying. Specifically, the shrinkage was minimal for SG15 (~3%), augmented significantly for SG25 (~29%) and much more pronounced for SG30 (~45%). Nevertheless, aerogel samples can withstand the stresses developed during the drying process, thus revealing the network-strengthening effect exerted by gelatin and GPTMS in this type of monolithic aerogel. For statistical analysis, 15 aerogel samples of each composition were obtained to verify the reproducibility of mechanical testing, swelling studies in PBS, biomineralization in SBF, and in vitro cell culture experiments.

**Figure 2:**
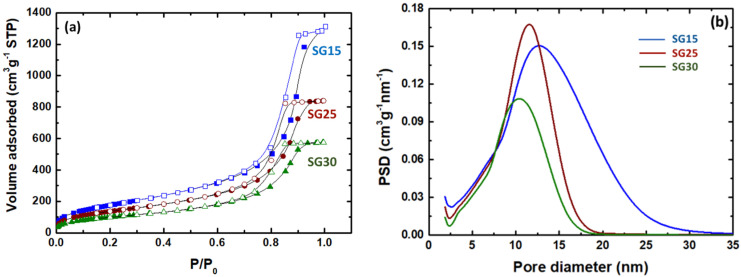
(**a**) N2–isotherms and (**b**) pore size distribution (PSD) of aerogels. (The full and open symbols represent the adsorption and desorption branches of each isotherm in Figure 2a, respectively).

N_2_-physisorption experiments revealed the effects of gelatin content on the structural features of the hybrids. A comparison of nitrogen isotherms for SG15, SG25 and SG30 aerogels are shown in Figure 2a. According to the IUPAC classification, all adsorption curves can be classified as type IV, accompanied by an H2 hysteresis loop typical of mesoporous networks [31] (Figure 2a). This is evidenced by the coexistence of the typical final saturation plateau of variable length at a high relative pressure and a long tail of the desorption isotherm directed toward a low relative pressure, indicating the existence of highly interconnected complex mesopore structures. The pore size distribution (PSD) curves of the hybrids were calculated from the desorption branch of the N_2_ isotherms using the Barrett–Joyner–Halenda (BJH) method [32] and are shown in Figure 2b. From this evaluation, it follows that the PSD narrows and the contribution of the smaller pores becomes more significant at a higher gelatin content.

In contrast, a wider PSD at a higher gelatin content was observed for a series of similar aerogels produced by co-gelation of gelatin and tetramethoxysilane (TMOS) without the addition of GPTMS or any other coupling agent [18]. In addition, the absence of a crosslinker caused these hybrids to develop an important distribution of micropores and macropores [33], which was not observed in our aerogel samples.

The corresponding textural parameters of SG15, SG25 and SG30 are listed in Table 2, all of which followed a decreasing trend with increasing gelatin content of the hybrids. The specific surface area (S_BET_) decreased from 651 m^2^g^−1^ to 361 m^2^g^−1^, the total pore volume (V_P_) from 1.98 cm^3^g^−1^ to 0.89 cm^3^g^−1^, and the mean pore diameter (d_pore_) was in the range of 10.8 nm to 8.6 nm for SG15 and SG30, in each case. This behavior was provoked by the progressive incorporation of gelatin and GPTMS into the polysiloxane network, which produced a highly crosslinked hybrid backbone, allowing mechanical strengthening and significant volumetric shrinkage of the aerogel monolith specimens, with a consequent increase in the bulk density, as shown in Table 2. A suitable explanation for the shift in the PSD versus smaller pore size at higher gelatin contents and the decrease in all textural parameters is that hybridization between the silica network and gelatin was enhanced by the presence of GPTMS. Therefore, the resulting hybrid material became more entangled and structurally regular as both gelatin and GPTMS increased until the organic phase reached its percolation threshold, presumably above a gelatin content of 25 wt. %. This amount corresponds to 40 wt. % gelatin relative to the silica produced from TEOS, which is the at the base of the siloxane-forming network of the hybrids, and therefore in agreement with theoretical and experimental observations previously made on the percolation theory of hybrid xerogels [34,35] and aerogels [36,37]. Further, although showing different trending behavior, the calculated textural parameters displayed similar values with those of different silica–gelatin aerogels obtained in supercritical CO_2_ without support of the crosslinker agent [18].

### 2.2. Thermogravimetric Analysis

**Figure 3:**
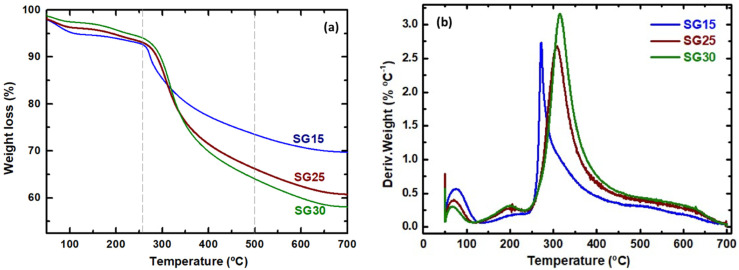
(**a**) Thermogravimetric curves and (**b**) derivative weight losses of the aerogels.

The gelatin content of the hybrid aerogels after processing was estimated using TGA from the weight loss in the range 250–500 °C (Figure 3a) according to a previously described method [1,26]. The results obtained were in good agreement with the nominal gelatin content used in the recipes, suggesting successful incorporation of the protein into the hybrid networks. The abrupt weight loss with a differential thermal analysis (DTA) endothermic peak occurring near 260 °C for the SG15 sample with low organic content (see Figure 3b) was attributed to fast gelatin decomposition. This is indicative of a weak hybridization between the inorganic and organic chains of the sample. Thus, SG15 was considered to be composed of small agglomerates of gelatin surrounded by a highly porous inorganic silica matrix. In contrast, the TGA curve profiles corresponding to the SG25 and SG30 aerogels revealed thermal stabilization of gelatin, which started to decompose at a higher temperature range of 310–320 °C.

### 2.3. Fourier-transform Infrared Spectral Analysis

**Figure 4:**
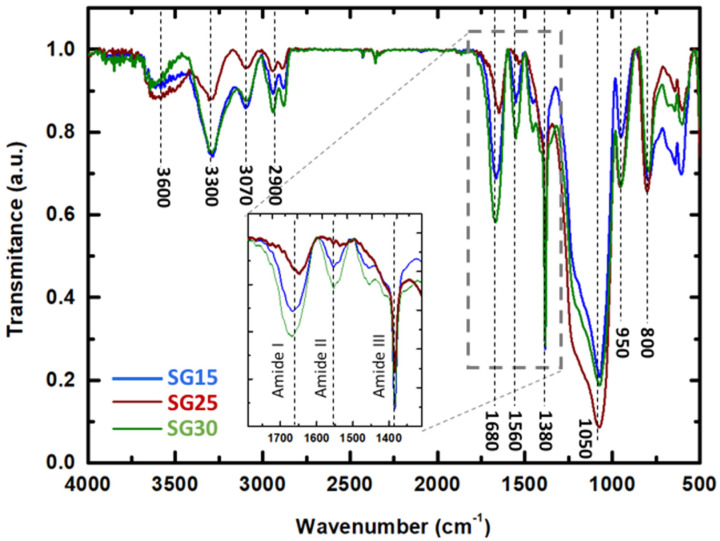
IR spectra of SG15, SG25, and SG30 aerogels. The inset shows a magnification of the intensity changes registered for the amide I, amide II, and amide III absorption bands.

The IR spectra of the SG15, SG25, and SG30 hybrid aerogels are shown in Figure 4, showing characteristic bands related to siloxane bond vibrations. Particularly at 800 and 1050 cm^−1^, absorption bands are attributed to symmetric and asymmetric stretching vibration of Si-O-Si, separately, [38] and the band at 950 cm^−1^ is assigned to the Si-O-H stretching vibration from hydrolyzed TEOS. Additionally, the bands at 1680 (C=O stretching), 1560 and 1380 cm^−1^ (N-H bending and C-N stretching) correspond to the vibrational modes of amides I, II, and III, respectively, which are typical of gelatin [2,39,40,41]. Other characteristic functional groups of gelatin present in the structure of the hybrids included amide band A at 3300 cm^−1^ (N-H stretching) and amide B at 3070 cm^−1^ (C-H stretching). Moreover, GPTMS showed two bands of absorbance close to 2850 and 2950 cm^−1^, corresponding to the vibration of the methylene and methyl groups. Additionally, the broad band centered at 3600 cm^−1^ originates from the O-H stretching vibration, thus revealing the hydrophilic character of the hybrids. The amide I, amide, and amide III absorption bands shifted to higher wavenumbers relative to their positions in the pristine gelatin, thus revealing strong covalent interactions with the crosslinker. In addition, the intensity decrease of the amide bands in SG25 relative to SG15 and SG30 in the range of 1350–1700 cm^−1^ signals a strong covalent interaction between gelatin and GPTMS [6], characteristic of functionalized hybrids [41]. Moreover, the band from the oxirane ring (908 cm^−1^) of the GPTMS precursor did not appear in any case, probably because it fully reacted with the carboxyl, hydroxyl, or amino organic functional groups present in the system. These features indicate the formation of a strengthened hybrid backbone, especially in the case of the SG25 aerogel.

### 2.4. Mechanical Properties

**Figure 5:**
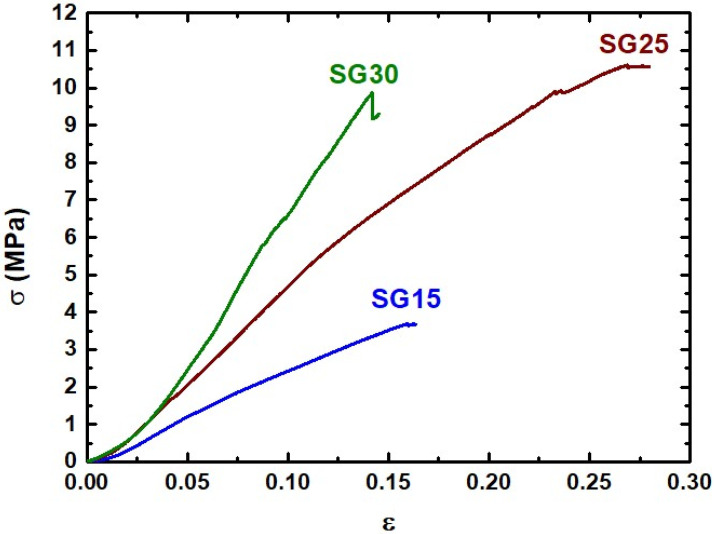
Stress–strain curves of the SG15, SG25, and SG30 aerogels obtained using uniaxial compression tests.

The stress–strain curves obtained from the uniaxial compression test are shown in Figure 5 for all hybrids immediately after being processed in an autoclave. These experiments provided a general view of the mechanical characteristics of the samples and information regarding Young’s modulus, compressive strength, and rupture strain. All samples displayed a comportment typical of elastic hybrid materials, with Young´s modulus being influenced by increasing gelatin content. It is worth noting the high initial stiffness exhibited by the SG30 stress–strain curve relative to the other samples. This can be induced by the prevalence of gelatin and GPTMS in the structure, which reacted with TEOS to form strong crosslinked hybrid networks that provoked strong volume contraction, resulting in the partial sealing of the mesopores, consequently increasing the rigidity of the material. Therefore, the SG30 aerogel can be considered as a composite-reinforced structure in which gelatin constitutes the matrix phase where silica clusters are embedded. In contrast, the SG15 sample exhibited the highest compliance response, thus revealing its structure, which was probably formed by an inorganic matrix of silica in which gelatin agglomerates were embedded. Additionally, as expected, the SG25 hybrid aerogel exhibited intermediate elastic behavior. However, it also displayed the highest rupture strain (above 27%), suggesting an interrelation between the structural and mechanical properties, which could be a sign of reaching the percolation threshold in this hybrid system, resulting in network strengthening. Table 3 lists the average Young´s modulus (varying from 31 to 78 MPa), compressive strength (in the range of 4–10 MPa), and maximum strain (between 14 and 27%), which were found to be much higher than the values obtained for similar aerogels in previous studies [6].

In addition, the samples were immersed in PBS and tested under uniaxial compression in a wet state to explore their reliability in a saline solution medium that provoked the appearance of fractures in a period ranging from a few seconds to several minutes, owing to the degradation of the hybrid networks. Consequently, a drastic reduction in all elastic parameters was observed. The data obtained are listed in Table 3.

### 2.5. Absorption Experiments

As observed and according to previous studies, water readily interacts with gelatin–silica aerogels, simultaneously hydrating their hybrid network, usually through capillary imbibition, and partially disintegrating their structure due to absorption by swelling, depending on the composition of the aerogel backbone [18]. However, without the incorporation of the GPTMS crosslinker, which forms strong covalent bonds between the organic and inorganic networks, disintegration of the materials may occur almost instantaneously after having been put in contact with liquids. To determine the absorption response of the aerogels, the PBS uptake abilities of SG15, SG25, and SG30 hybrids were examined in terms of their swelling ratio and absorption kinetics. All experiments were performed in triplicate to guarantee reproducibility.

#### 2.5.1. Swelling Ratio

Swelling ratios of 2.32, 3.32, and 3.04 were calculated from Equation (1) for SG15, SG25, and SG30 aerogels, respectively (Table 4). The role of gelatin in favoring PBS absorption was evidenced in our study, especially by the absorption responses of SG25 and SG30, which displayed a significant increase in liquid uptake compared to the original porous volume of the samples, involving the action of swelling when this ratio exceeded unity.

#### 2.5.2. Swelling Kinetics

**Figure 6:**
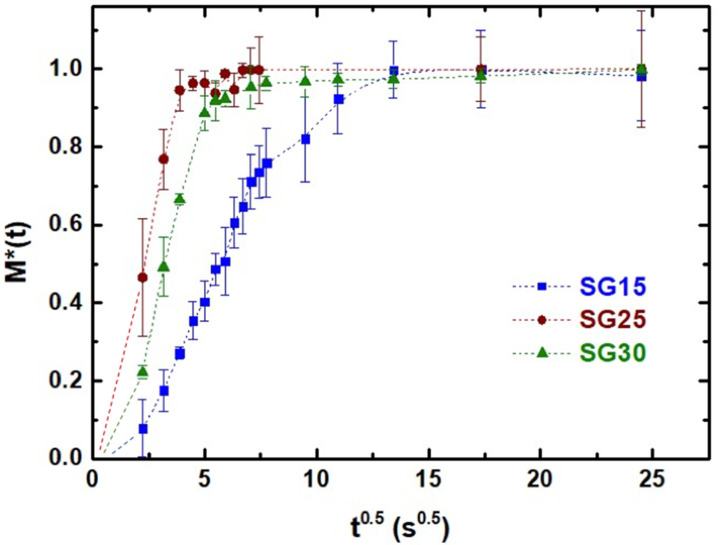
Normalized weight gain (M*(t) = M(t)/M∞) for PBS uptake in SG15, SG25 and SG30 versus the square root of time. Linear fittings were performed for up to 80% of the processes for SG15, SG25, and SG30. Correlation values of R^2^ > 0.984 in all cases. The dashed lines represent the eye guide.

The uptake of PBS by the aerogels occurred almost instantaneously and a saturation level of M∞ was achieved within seconds. In addition, boundaries separating the highly swollen region from the dry region were not detected, indicating Fickian diffusion [42]. Following, the effect of the gelatin content on the absorption kinetics of the three hybrid samples was evaluated by studying the normalized weight gain, specifically the ratio M(t)/M∞ against square-root of t (Figure 6) in PBS at room temperature. This revealed that PBS diffused faster in SG25 and SG30 than in the SG15 aerogel hybrids. Additionally, it was confirmed that the normalized weight gain M(t)/M∞ vs. t^0.5^ was dominated by a Fickian diffusion mechanism [43], demonstrating that the PBS uptake was proportional to the square root of time, fitting the linear trend (R^2^ > 0.989) well before reaching saturation (M(t)/M∞ ≈ 0.8). The corresponding linear-fitting data for Fick´s law are presented in Table 4. These results are in accordance with those of previous studies on similar hybrid mesoporous matrices that incorporated chitosan as a biopolymer instead of gelatin [10,44].

### 2.6. In Vitro Bioactivity in SBF

**Figure 7:**
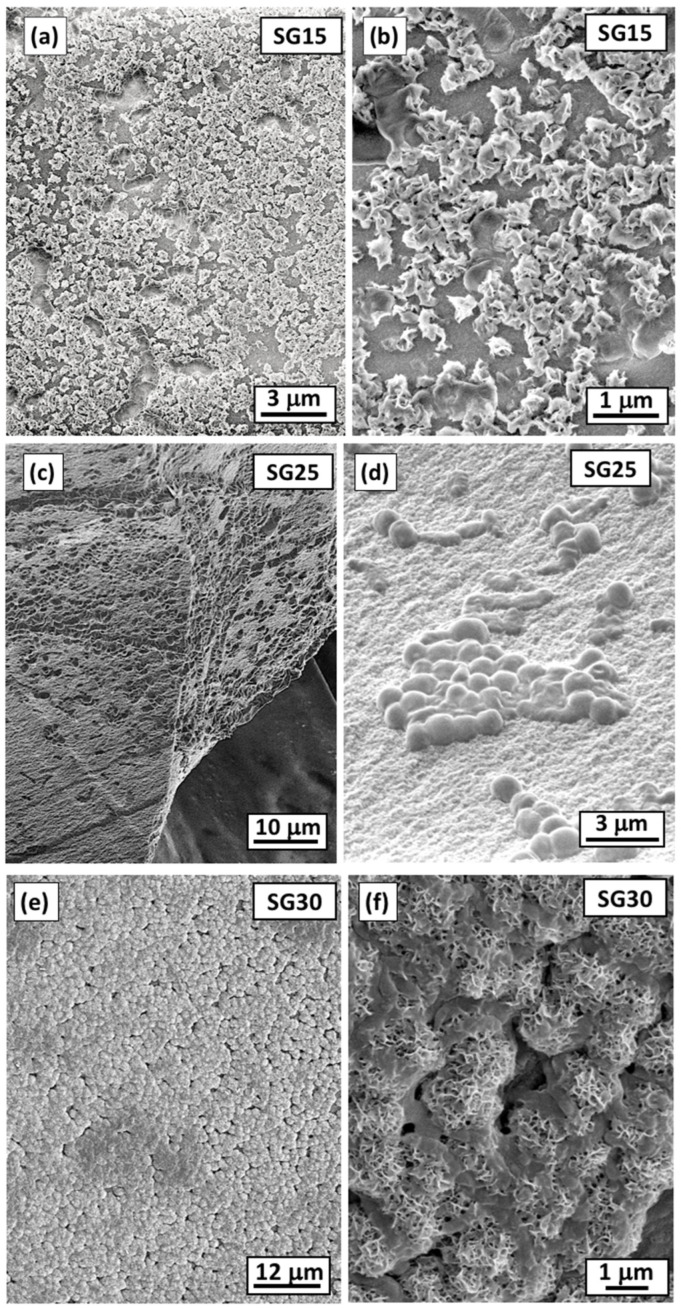
SEM micrographs of the surfaces of (**a**,**b**) SG15, (**c**,**d**) SG25, and (**e**,**f**) SG30 aerogels after soaking in SBF for 21 days at 37 °C exhibiting a bioactive response through the formation of a hydroxyapatite (HAp) layer at different stages of crystal growth.

Micrographs of the hybrids after testing their bioactivity in SBF are shown in Figure 7. All samples presented an apatite-like structure on their surfaces 21 days after soaking in SBF, which was formed through the interaction of calcium and phosphate ions from the surrounding media. The morphology and size of the mineralized products were characterized using scanning electron microscopy (SEM) and its composition with elemental energy-dispersive spectroscopy (EDS) analysis. SEM micrographs revealed that for the selected soaking period, the morphologies of the aggregates deposited on the surfaces were influenced by the gelatin content of the aerogels, resulting in the formation of mineralized hydroxyapatite (HAp) at different stages of the nucleation-growth process, covering approximately the entire aerogel surface [45]. For example, the distribution of submicrometric aggregates of undefined morphology deposited on the SG15 surface (Figure 7a,b) was composed of Ca and P with a 1.12 molar ratio, indicating the formation of a calcium–phosphate (CaP) product that was found at its initial crystal growth stage. Therefore, it was still far from the ideal stoichiometric HAp molar ratio (1.67). Similarly, typical HAp microsized spherulites, measuring approximately 1 μm in diameter, were deposited on the surface of the SG25 aerogel (Figure 7c,d). The corresponding Ca/P ratio was 1.35, indicating its progression towards HAp formation. Finally, the SG30 aerogel presented the highest Ca/P ratio (1.38) but was still far from the ideal Ca/P value. However, a layer composed of spherulites completely covering the surface was formed in this case (Figure 7d,e), an effect attributed to the high gelatin content that promotes biomineralization [28,46].

### 2.7. Live Dead Assays, Cell Morphology and Cytoskeletal Changes

**Figure 8:**
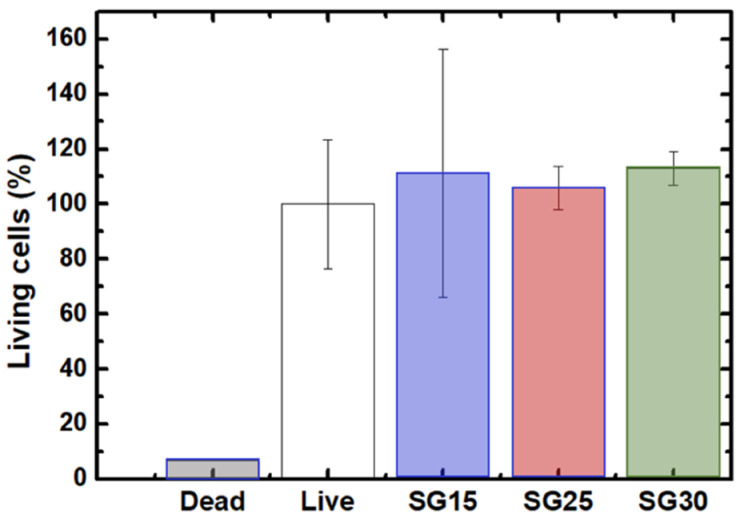
Quantitative data for live/dead assay after 1 week in culture.

**Figure 9:**
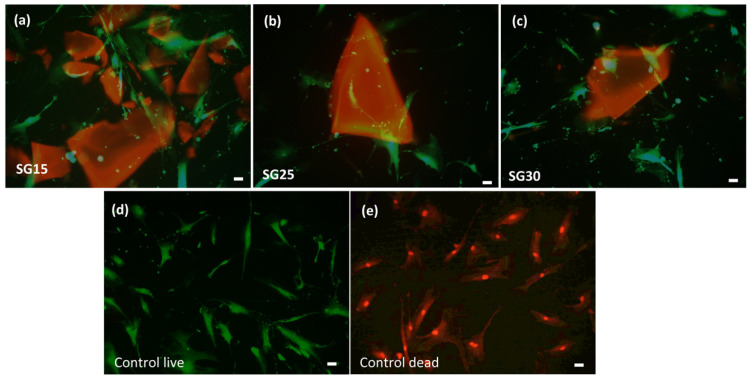
Live/dead staining of HOB^®^ cells after one week of culture, grown in the presence of SG15 (**a**), SG25 (**b**) and SG30 (**c**) aerogels. Live and dead controls showing cells growing without materials are shown in (**d**) and (**e),** respectively. Images were acquired in the fluorescence and Nomarski modes using a Leica DMIL LED inverted microscope. Live cells appear green and the nuclei of dead cells are stained red. In the merged images, the aerogels appear soft red. Scale bar: 20 μm.

**Figure 10:**
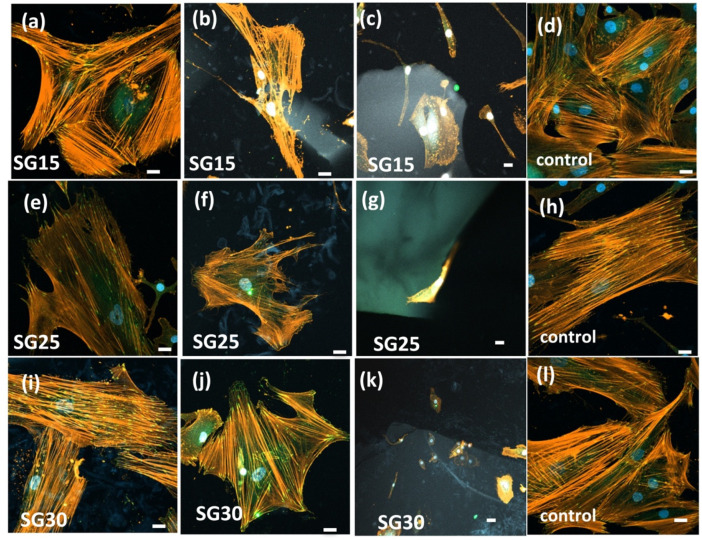
Representative images of HOB^®^ osteoblasts after one week of culture, grown in the presence of SG15 (**a**–**c**), SG25 (**e**–**g**), and SG30 (**i**–**k**) aerogels, and control groups grown on glass (**d**,**h**,**l**). Images were acquired using a confocal microscope, after immunolabelling the actin cytoskeleton with rhodamine phalloidin (red fluorescence) and vinculin (green fluorescence) to detect focal adhesions. Blue—DAPI-labelled nuclei. Unless otherwise specified, the scale bar is 20 μm.

The osteoblast response in the presence of the aerogels revealed no cytotoxicity after live/dead assays, without significant differences between the experimental groups and positive controls. Cell viability at seeding was up to 98% in all groups and after one week, all the experimental groups differed significantly from the negative control (Figure 8). Cell polarization towards the particulate material was evident from the first 24/48 h. As shown in Figure 9, after one week, osteoblasts elongated towards and colonized particulate aerogels with very few or no dead cells in the wells. Filopodial and lamellipodial emissions approaching silica–gelatin fragments were evident and also appeared between the neighboring cells, mostly in SG30 groups. Although cell emissions in the SG15 and SG25 groups were shorter than those in SG30, cell colonization of the biomaterial was successful in all three aerogel materials (Figure 10).

Immunolabeling of osteoblasts with rhodamine phalloidin revealed that the actin cytoskeleton presented a double organization depending on the growth surfaces, which was also related to the successful development of focal adhesion (FA) points identified after immunolabeling with vinculin. Vinculin is a force-carrying component between adhesive sites and the cytoskeleton and localizes with areas of high force during leading edge protrusion [47,48,49]. The vinculin head domain promotes integrin clustering and increases the residence time of mature FAs in spread cells [50,51,52]. In osteoblasts grown on aerogel samples, the actin cytoskeleton leads to stress fiber bundles’ organization and successful FA maturation. The spatiotemporal regulation of actin cytoskeleton tension mediated by FAs is recognized to be of capital importance in modulating cell migration, with a main role as an anchorage reinforcement point for organic extracellular matrix remodeling for bone regeneration. Force-mediated FA signaling together with actin bundles’ organization in stress fibers regulates both cell proliferation and differentiation [50,53,54]. Actin cytoskeleton immunolabeling in HOB^®^, after 1 week in culture, revealed that the osteoblasts successfully settled on silica–gelatin samples, stress fibers formed a well-developed actin cytoskeleton and FAs appeared to be widely distributed on the tips of stress fibers. Both stress fiber and FAs were more abundant in osteoblasts grown in the presence of SG30, as shown in Figure 10. In SG15 and SG25, although some stress fibers can be appreciated, the actin cytoskeleton is mostly organized in thin fibers, disposed to the periphery, and cells remain elongated and polarized to particulate silica–gelatin samples, with filopodial and lamellipodial emissions even up to one week in culture.

**Figure 11:**
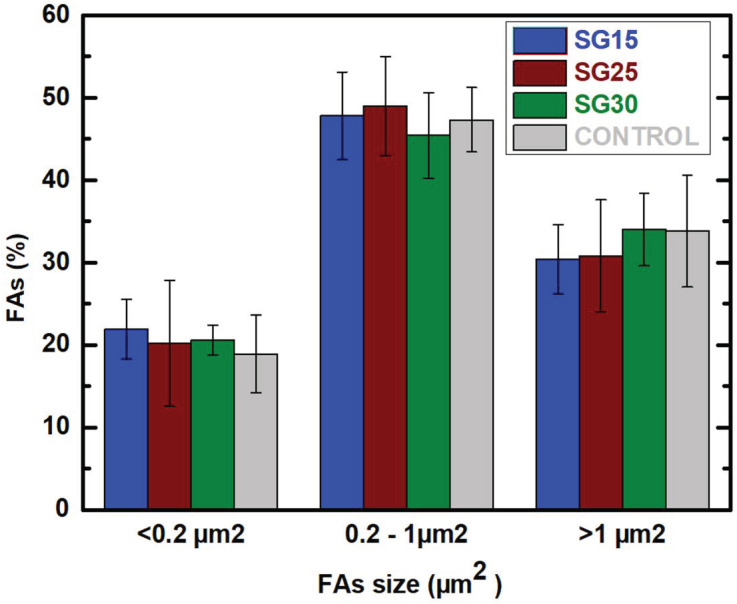
Percentage of FAs according to size in HOB^®^ cells grown on the described SG15, SG25 and SG30 aerogels after one week of culture. One-way analysis of variance. Statistical significance was defined as *p* < 0.05.

Cell morphology in these groups matches with cell migration features, and this fact is reinforced when focal adhesion size and distribution are analyzed [10,47,55,56,57]. FAs are formed during initial cell adhesion and constantly assemble and disassemble during cell movement thereafter, serving as mechano-sensors that recognize both biochemical and biophysical features [58]. Vinculin immunolabelling revealed how mature FAs sized > 1 μm^2^ predominate in SG30 groups, while small and medium sized predominate both in SG15 and SG25 (Figure 11) although no significant differences were found between those groups. Maturation of FAs is essential for adequate adhesion to the surface and subsequent transduction of outside–in and inside–out signaling, as well as the regulation of signaling pathways, leading to transcription factors that can contribute to the activation of cell processes leading to cell proliferation, migration and differentiation [58].

**Figure 12:**
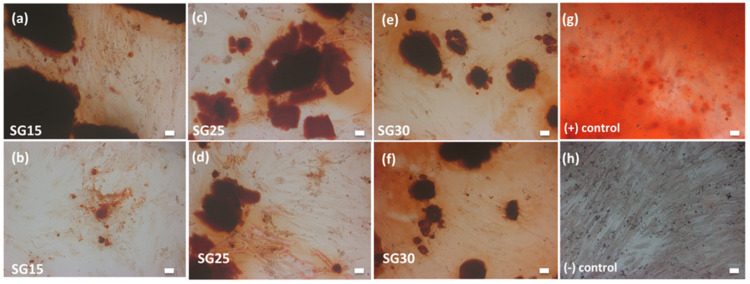
Alizarin red staining (red) reveals the effect of aerogels SG15 (**a**,**b**), SG25 (**c**,**d**), SG30 (**e**,**f**). Positive and negative controls are shown in (**g**,**h**), respectively. Scale bar represents 20 μm in all cases.

**Figure 13:**
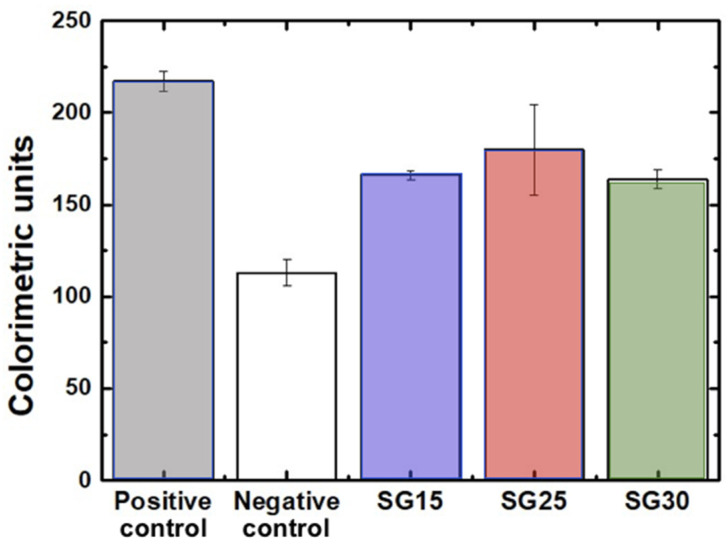
Alizarin red staining. Quantitative measurements for aerogels SG15, SG25, SG30 and controls. One-way analysis of variance. Statistical significance was defined as *p* < 0.05.

Alizarin red staining (ARS) is a well-established method for characterizing a mineralized matrix due to the differentiation of osteogenic lineage cells and allows the simultaneous evaluation of mineral distribution and inspection of structures through optical microscopy [47]. After 28 days in culture, the staining of the constructs with ARS revealed a deeply stained mineralized layer with calcium deposits within cells and the extracellular matrix, clearly visualized under 10x magnification (Figure 12). Mineralization assays on the three aerogels showed significant differences (*p* < 0.05) with osteoblasts grown on glass used as negative control (Figure 13).

The surface topography of implantable biomaterials is especially critical for guiding cellular behavior, such as adhesion, spreading, and cell movement [59]. Adequate directional migration and correct positioning of cells on biomaterials are the principles for promoting wound healing and tissue regeneration [58]. During migration, cells respond to chemical and mechanical cues by adapting their shape, dynamics and adhesion to the extracellular matrix (ECM) [60,61]. Quantitative data from morphometric analysis of the silica–gelatin samples revealed that osteoblasts on SG30 presented the biggest area and perimeter (*p* = 0.0296) and were less round and solid than those on the control. In addition, the SG15 groups presented low roundness and solidity values when compared to controls (*p* = 0.029) due to their elongated and migration-like cell morphology. These results appear on Table 5.

These findings indicate that the silica–GPTMS–gelatin mesoporous aerogels have excellent biocompatibility and promising bone formation ability. The excellent cell response observed can be attributed not only to the topographical effects of the nanostructured surfaces [62] but also to the following features of the aerogels: mesopores [63], high BET surface area [64], and probably to the physiological environment provided by the biodegradation of the hybrid matrix, which provides a biocompatible and bioactive surface for cells [65]. The synergistic effect of all these parameters was observed to promote the biomimetic behavior of silica-based aerogels with gelatin contents up to 30 wt. %. However, the effect of higher gelatin weight percentages and the resultant structural features of the new hybrids, as well as the details of their biodegradation, on the osteoblast behavior of silica–GPTMS–gelatin aerogels remains unclear and needs to be determined in future studies.

## 3. Conclusions

In conclusion, silica–GPTMS–gelatin mesoporous aerogels with 15, 25, and 30 wt. % gelatin content obtained through sol–gel processing in combination with supercritical drying in CO_2_ can be used as biocompatible materials for bone tissue engineering. Based on the observed structural properties and mechanical behavior, this system offers an experimental platform for studying the formation of hybrid materials with various ratios of organic and inorganic components. In this scenario, 25 wt. % gelatin corresponds to the critical concentration of the relative organic content at which qualitative and quantitative changes occurred. Moreover, these aerogels showed hydrophilic behavior and the capacity to induce nucleation and control the growth of a bioactive layer in SBF formed by HAp of different sizes (<1 μm) and morphologies, depending on the gelatin content, which could effectively contribute to bone regeneration. The live/dead assay revealed non-cytotoxic characteristics of the samples. Furthermore, they promoted cell growth, adhesion, and mineralization, with time-dependent focal adhesion maturation and cytoskeletal reorganization, mainly in samples with higher gelatin content. Owing to the versatility of the particulate format of hybrid biomaterials, easy sterilization, and manipulation, a wide range of applications open up avenues for bone regeneration in dentistry, traumatology, and orthopedic surgery, both in isolated form and as base matrices for bioactive reinforcement phases. Once tested in human cell lines, our research interest points to the primary mesenchymal stem cell differentiation response to the described biomaterials as a preliminary step for animal studies.

## 4. Materials and Methods

### 4.1. Materials

Tetraethylorthosilicate (TEOS, 99%) and chloride acid (HCl) (37%) were obtained from Alfa Aesar (Haverhill, MA, USA). Gelatin (type A porcine, gel strength 300) and 3-glycidoxypropyltrimethoxysilane (GPTMS, >98%) were purchased for Sigma Aldrich (St. Louis, MO, USA). Absolute ethanol (99.5%) was purchased from Panreac and acetid acid (Reagent Grade) was purchased from Scharlau (Barcelona, Spain). HOB^®^ human osteoblasts, fetal calf serum, osteoblast growing medium and osteoblast mineralization medium were purchased from Promocell (Heidelberg, Germany). Paraformaldehyde, PBS, Triton FITC conjugate were all purchased from Sigma, (St. Louis, MI, USA) and Vectashield^®^ (Vector Laboratories, Burlingame, CA, USA). Alizarin red staining solution was purchased from Fisher Scientific, (Waltham, MA, USA).

### 4.2. Preparation of Sols and Aerogels

**Figure 14:**
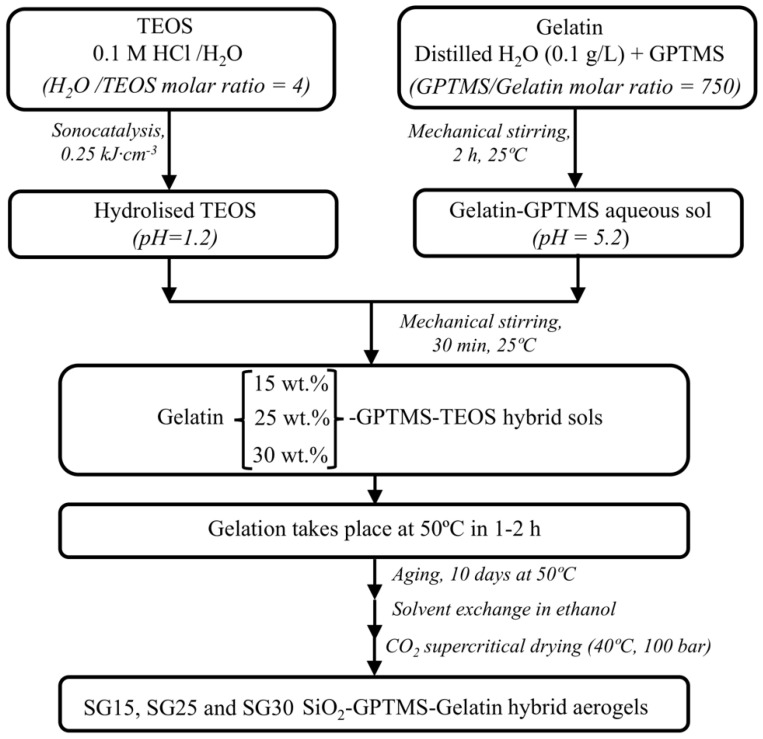
Scheme of the synthesis procedure for silica/GPTMS/gelatin hybrid aerogels. GPTMS/gelatin of 750 was kept constant in all cases and samples were named SGx, where x = 15, 25, 30, indicating that the gelatin wt.% referred to the theoretical silica content obtained from TEOS and GPTMS precursors.

The samples were prepared using a one-step sol–gel method, in which TEOS, as the silica source, was hydrolyzed under acidic conditions with an aqueous HCl/TEOS molar ratio of 4, assisted by high-power ultrasound. In addition, an aqueous gelatin sol (pH 5.2) was separately functionalized with GPTMS for 2 h at room temperature while maintaining a constant gelatin/GPTMS molar ratio (C-factor) of 750. The functionalized gelatin–GPTMS aqueous sol (pH 5.2) was used as a precursor by adding appropriate amounts of hydrolyzed TEOS (pH 1.2) to produce hybrid sols with approximately 15, 25 and 30 wt. % gelatin content, in relation to the total silica content that can be obtained from both the TEOS and GPTMS silane precursors. These sols were mixed for 30 min at room temperature, where the network-forming condensation reaction between hydrolyzed TEOS and functionalized gelatin began. The estimated gelation time varied from 60 min to 120 min by decreasing the organic content. The hybrids were aged at 50 °C for 10 days, followed by an ethanol wash for another 5 days to eliminate all impurities. Finally, all the gels were dried under supercritical drying in CO_2_ at 90 bar and 40 °C and named SGx, where x indicates the wt. % of gelatin. A schematic of the complete synthetic procedure is shown in Figure 14. Normally, 15 aerogel monolith cylindrical samples of each composition were prepared in order to perform a detailed study and to ensure reproducibility for mechanical testing, absorption capacity and absorption kinetics in phosphate-buffered saline (PBS), biomineralization, and cell culture.

### 4.3. Physical and Textural Characterization

The geometric apparent densities of the samples were obtained by measuring the mass using a microbalance (precision of ± 0.1 mg) and the sizes of the monolithic samples were measured using a sliding caliper.

The textural parameters of the hybrid samples (BET surface area, pore volume, and pore size distribution from BJH standard model) were determined using nitrogen adsorption at 77 K on an ASAP 2020 analyzer (Micromeritics, Norcross, GA, USA) equipped with a pressure transducer resolution of 10^−4^ mmHg. Before the measurements, the samples were degassed under vacuum while heating at 120 °C for 4 h so that the experiment could be performed in an air-dry state.

### 4.4. Thermal Characterization

The thermal stability of the aerogels was determined using thermogravimetric analysis (TGA) by monitoring their weight changes in the range of 50–700 °C under an air atmosphere at a constant heating rate of 10 °C min using a TGA Q50 apparatus (TA Instruments, New Castle, DE, USA).

### 4.5. FTIR Spectroscopy

FTIR spectra were obtained using a Bruker Tensor 37 spectrophotometer (Billerica, MA, USA) operating at room temperature with a resolution of 4 cm^−1^ and 100 scans in the region from 500 to 4000 cm^−1^. The samples were ground into a fine powder, mixed with KBr, pressed into a self-supporting wafer that was stored for 24 h at 60 °C, and further placed on a sample holder for spectrum measurement.

### 4.6. Mechanical Properties

The mechanical properties of the samples were studied using uniaxial compression (Shimadzu AG-I Autograph, Kyoto, Japan) with a load cell of 5 kN for dry samples and 500 N for samples immersed in PBS. Cylindrical specimens, measuring 16–20 mm in height and 8–10 mm in diameter, were used in accordance with ASTM D7012 (h = 2D). Young´s modulus was calculated from the initial tangent of the stress–strain curve. The maximum compressive stress and maximum strain were determined from the maximum deformation before failure.

### 4.7. Absorption Experiments in PBS

Absorption experiments were conducted in triplicate using a previously described procedure [10]. Briefly, a cylindrical hybrid (5 mm high and 8–10 mm diameter) was immersed in PBS at room temperature. The absorption process was studied by measuring the time evolution of the relative weight gain M(t) until saturation was reached, defined as the relative increase in the weight of the sample owing to PBS absorption per unit weight of the dry sample, and was calculated according to Equation (1):

(1) $$M\left(t\right)=\frac{W_{t}-W_{d}}{W_{d}}$$

where Wt is the actual weight in the swollen state for a given time and Wd is the weight of the dry sample. The weight gain value at the saturation point (namely for t ⟶ ∞), gives the corresponding absorption capacity or swelling ratio (SR), which accounts for the PBS uptake through all possible absorption mechanisms (mainly spontaneous imbibition and swelling). The swelling ratio was also assessed as the ratio of the volume of absorbed PBS to the original porous volume of the sample, revealing the presence of swelling if this ratio exceeded unity.

Finally, for kinetic studies, the absorption behavior was described by the normalized weight gain M* (t) =M(t)/M∞, where M∞ is the weight gain of the sample after saturation.

### 4.8. Biomineralization Experiments

Biomineralization was studied by immersing 5 mm length × 8 mm diameter aerogel pellets in 20 mL simulated body fluid SBF [66] in polyethylene flasks and evaluating the formation of hydroxyapatite (HAp) at the surface during 21 days of soaking at 37 °C, while the SBF fluid was exchanged weekly. The samples were removed once a week from the buffer solution, carefully washed with Milli-Q water (Millipore Sigma, Burlington, MA, USA), and stored at 50 °C under ambient pressure. The surface morphologies of the samples after different soaking intervals were examined using a FEI Nova NanoSEM 450 (FEI, Morristown, NJ, USA) (resolution 1.4 nm) equipped with a Bruker SDD-EDS detector, which was used to determine the Ca/P composition across the specimen surface.

### 4.9. Cell Culture

HOB^®^ cells were seeded onto aerogels under sterile conditions. Once confluent, the cells were detached and analyzed for cell viability (Luna^®^ Cell Counter, Invitrogen). The cells used in the experiments did not exceed ten population doublings. Prior to cell seeding, the aerogels were sterilized in a clinically standardized B autoclave (under European standard DIN EN ISO 13,060 recommendations) and then placed in Mattek^®^ glass-bottom wells. A drop of 50 μL of cell suspension at a density of 15,000 HOB^®^ cells/cm2 was added to each sample and incubated for 30 min under humid conditions at 37 °C and 5% CO_2_ to ensure optimal cell attachment and avoid dispersion. The wells were then filled with OGM^®^ supplemented to a final concentration of 0.1 mL/mL fetal calf serum at 37 °C and 5% CO_2_, and incubated during the experiment. The test groups were SG15, SG25 and SG30. HOB^®^ cells grown on glass were used as controls.

### 4.10. Live/Dead Cell Assay

Live/dead cell assay was performed to evaluate the viability and cytotoxicity in HOB cells grown on the hybrid aerogels. After incubation for seven days, the constructs were rinsed twice with PBS and stained with calcein-AM (0.5 μL/mL) in PBS and ethidium homo-dimer-1 (EthD-1) (2 μL/mL) in PBS to visualize live and dead cells, respectively. Negative controls were treated with 70% ethanol for 30 min before labeling. Images of the cell/scaffold constructs were acquired using a Leica DMIL LED inverted microscope in the Nomarski and fluorescence modes.

### 4.11. Cell Morphology and Spreading

HOB^®^ cells were examined daily using a phase–contrast microscope to evaluate the initial adhesion phase to the surfaces (cell morphology, alignment, distribution, and spreading) prior to immunolabeling. Both fluorescence and confocal examination were combined, when possible, with fluorescence and Nomarski modes to acquire both material and growing cells.

### 4.12. Actin Cytoskeletal Organization

Osteoblasts grown in the presence of aerogels were immunolabeled with rhodamine-phalloidin and vinculin to assess actin cytoskeleton changes and FA development, after one week of culture. After washing with PBS (pH 7.4), the cells were fixed with 3.7% paraformaldehyde at room temperature, washed, permeabilized with 0.1% Triton x-100, washed, and preincubated with 1% bovine serum albumin in PBS for 20 min prior to immunolabeling. After 20 min, the samples were rinsed prior to mounting with Vectashield^®^. At least five samples of each type were analyzed in each experiment. The test groups were SG15, SG25, SG30. HOB^®^ cells grown on glass were used as controls. The focal adhesion size and location were measured in at least 10 cells on each substrate.

### 4.13. Confocal Examination

At least five samples were analyzed in each group to assess cytoskeletal organization, focal adhesion number and development, and cell morphology in the test groups. At least 10 cells were analyzed per sample, and images were collected and processed using an Olympus BX40 microscope with DP73 camera imaging software. The samples were exposed for less than 5 min to avoid photobleaching and to the lowest laser power that could produce a fluorescent signal with one Airy unit pinhole. Images were acquired at a resolution of 1024 × 1024 pixels.

### 4.14. Image Analysis

Sample images were collected as frames obtained at 40x magnification and processed using Image J software (NIH, http://rsb.info.nih.gov/ij accessed on 12 January 2023). At least 40 regions of interest (ROIs) were measured for quantitative analysis. All ROIs were selected based on the following criteria: well-defined limits, clear identification of the nucleus, and absence of intersection with neighboring cells. The number and size of FAs were assessed in the sample groups. The area, perimeter, roundness, circularity, and aspect ratio were analyzed as shape variables.

### 4.15. Mineralization

Osteoblast mineralization medium was used for 28 days to induce mineralization in the silica–gelatin–HOB ^®^ constructs. HOB^®^ cells cultured in osteoblast growth medium were used as a negative control, and cells grown only in osteoblast mineralization medium without aerogels were used as positive mineralization controls. The cells and constructs were incubated at 37 °C and 5% CO_2_, and the medium was changed three times per week.

### 4.16. Detection of Mineralization

Mineralization capability was evaluated after 28 days of culture using an alizarin red staining solution. The cells and constructs were fixed with 4% paraformaldehyde for 1 h at room temperature, washed three times with PBS, stained with 2% alizarin red solution, and rinsed with PBS. Calcium deposits within the cells and extracellular matrix were visualized under a fluorescence microscope (magnification 10x) and processed using Image J software.

### 4.17. Statistical Analysis

All experiments were performed in triplicate unless otherwise stated. All data were analyzed using SPSS and presented as mean ± standard deviation. Once normality and homoscedasticity were confirmed, the difference between the mean values was analyzed using one-way analysis of variance and the Brown–Forsythe and Games–Howell tests.

## Figures and Tables

**Table 1:** Nominal compositions of the prepared hybrid aerogel materials.

Sample Code	Gelatin (g)	SiO_2_ from TEOS (g)	SiO_2_ from GPTMS ^1^ (g)	Gelatin Content (wt. %)
SG15	0.8	4.1	0.4	15.1
SG25	1.6	4.1	0.6	25.4
SG30	2.4	4.1	1.3	30.7

^1^ GPTMS/gelatin molar ratio of 750 was kept constant in all cases.

**Table 2:** Bulk density, volume shrinkage and textural parameters (S_BET_ specific surface area, specific porous volume and pore size) were measured for the different hybrid aerogels obtained as a function of the gelatin content.

Sample Code	Bulk Density, ρ_a_ (g cm^−3^)	Volume Shrinkage (%)	S_BET_ (m^2^g^−1^)	Pore Volume, V_p_ (cm^3^g^−1^)	Pore Size ^1^, d_pore_ (nm)
SG15	0.41 ± 0.01	3.3 ± 1.0	651 ± 11	1.98	10.8
SG25	0.56 ± 0.01	28.8 ± 2.7	482 ± 15	1.30	9.20
SG30	0.69 ± 0.03	44.6 ± 1.0	361 ± 10	0.89	8.60

^1^ Pore size (d_pore_) stands for the average typical pore size. Errors represent the standard deviation from three replicate measurements.

**Table 3:** Elastic parameters of the hybrid aerogels tested under uniaxial compression, as obtained (in the dry state) and submerged in PBS (in the wet state).

Sample	Young’s Modulus (MPa)		Compressive Strength (Mpa)		Maximum Compressive Strain (%)	
	Dry	Wet	Dry	Wet	Dry	Wet
SG15	30.81 ± 1.72	3.71 ± 0.33	3.69 ± 0.25	0.33 ± 0.01	16.29 ± 0.21	4.55 ± 0.44
SG25	45.74 ± 3.26	--	9.67 ± 1.66	--	27.67 ± 0.47	--
SG30	78.55 ± 5.11	1.65 ± 0.36	9.90 ± 2.35	0.10 ± 0.01	14.06 ± 1.35	4.08 ± 0.53

**Table 4:** Swelling ratio and parameters from the linear fitting performed for up to 80% of the absorption process, according to Fick´s diffusion model, for PBS uptake through SG15, SG25, and SG30 aerogels.

Samples	Swelling Ratio	Linear Fit: (M(t)/M∞) = a + bt^0.5^		
		a	b	R^2^
SG15	2.32 ± 0.11	−0.22 ± 0.03	0.13 ± 0.02	0.9948
SG25	3.42 ± 0.21	−0.18 ± 0.07	0.29 ± 0.02	0.9893
SG30	3.04 ± 0.14	−0.29 ± 0.06	0.24 ± 0.02	0.9847

**Table 5:** Shape variables. Data quantified in Image J. Significant differences between groups are denoted by * (*p* < 0.05).

Sample	Area (μm^2^)	Perimeter (μm)	Circularity	Aspect Ratio	Roundness	Solidity
SG15	16271 ± 21547	958 ± 786	0.189 ± 0.089	3.63 ± 1.74	0.33 ± 0.13 *	0.64 ± 0.21
SG25	6229 ± 7087 *	459 ± 349 *	0.360 ± 0.178	2.26 ± 0.96	0.50 ± 0.18	0.71 ± 0.08
SG30	29,077 ± 44,946 *	1231 ± 1166 *	0.207 ± 0.157	3.13 ± 2.03	0.42 ± 0.23 *	0.65 ± 0.16
Control	5302 ± 1268	630 ± 21 *	0.166 ± 0.028	3.02 ± 1.87	0.87 ± 0.08 *	0.68 ± 0.05

## Data Availability

Not applicable.
